# Ppbv-Level Ethane Detection Using Quartz-Enhanced Photoacoustic Spectroscopy with a Continuous-Wave, Room Temperature Interband Cascade Laser

**DOI:** 10.3390/s18030723

**Published:** 2018-02-28

**Authors:** Chunguang Li, Lei Dong, Chuantao Zheng, Jun Lin, Yiding Wang, Frank K. Tittel

**Affiliations:** 1 College of Biological and Agricultural Engineering, Jilin University, Changchun 130022, China; lcg@jlu.edu.cn; 2 Department of Electrical and Computer Engineering, Rice University, 6100 Main Street, Houston, TX 77005, USA; zhengchuantao@jlu.edu.cn (C.Z.); fkt@rice.edu (F.K.T.); 3 State Key Laboratory of Quantum Optics and Quantum Optics Devices, Institute of Laser Spectroscopy, Shanxi University, Taiyuan 030006, China; 4 National Engineering Research Center of Geophysics Exploration Instruments, College of Instrumentation & Electrical Engineering, Jilin University, Changchun 130061, China; lin_jun@jlu.edu.cn; 5 State Key Laboratory on Integrated Optoelectronics, College of Electronic Science and Engineering, Jilin University, Changchun 130012, China; ydwang@jlu.edu.cn

**Keywords:** laser sensors, infrared spectroscopy, semiconductor quantum cascade lasers

## Abstract

A ppbv-level quartz-enhanced photoacoustic spectroscopy (QEPAS)-based ethane (C_2_H_6_) sensor was demonstrated by using a 3.3 μm continuous-wave (CW), distributed feedback (DFB) interband cascade laser (ICL). The ICL was employed for targeting a strong C_2_H_6_ absorption line located at 2996.88 cm^−1^ in its fundamental absorption band. Wavelength modulation spectroscopy (WMS) combined with the second harmonic (2f) detection technique was utilized to increase the signal-to-noise ratio (SNR) and simplify data acquisition and processing. Gas pressure and laser frequency modulation depth were optimized to be 100 Torr and 0.106 cm^−1^, respectively, for maximizing the 2f signal amplitude. Performance of the QEPAS sensor was evaluated using specially prepared C_2_H_6_ samples. A detection limit of 11 parts per billion in volume (ppbv) was obtained with a 1-s integration time based on an Allan-Werle variance analysis, and the detection precision can be further improved to ~1.5 ppbv by increasing the integration time up to 230 s.

## 1. Introduction

Ethane (C_2_H_6_) is one of the most abundant non-methane hydrocarbon in the atmosphere that strongly affect both atmosphere chemistry and the climate [1,2]. C_2_H_6_ usually originates from fossil fuel and biofuel consumption. Hence, C_2_H_6_ detection at low concentration levels is very important in environmental monitoring [3,4]. Furthermore, ultra-sensitive detection of ethane can be applied to breath analysis as a non-invasive medical diagnostic method for identifying and monitoring C_2_H_6_ concentration levels in the exhaled breath of patients, such as the identification of asthma by the detection of C_2_H_6_, which is generated by oxidative stress [5], the measurement of exhaled C_2_H_6_ as a direct biomarker of schizophrenia due to increased *n* − 3 lipid peroxidation [6], and, in the analysis of lung cancer, by detecting C_2_H_6_ as a marker of oxidative stress [7].

Gas detection techniques based on optical absorption have many advantages, such as fast response time, high gas selectivity, high measurement precision, no requirement for any sample pretreatment, and minimal drift. Hence, the tunable diode laser absorption spectroscopy (TDLAS) technique that employs a multi-pass gas cell (MGC) [8], the photo-acoustic spectroscopy (PAS) technique that employs a broadband microphone [9], the dispersion spectroscopy technique [10], and the photothermal interferometry technique [11] have been widely used in recent years. However, sensors based on MGC and PAS are large in size. Quartz-enhanced photoacoustic spectroscopy (QEPAS) [12] is an alternative approach, instead of conventional photo-acoustic spectroscopy, which utilizes a millimeter sized piezoelectric quartz tuning fork (QTF) as an acoustic wave transducer to detect photo-acoustic excitation induced by a modulated laser source absorbed by the gas target [13]. A high Q-factor (>10,000) and a ~32.7 kHz resonance frequency of the QTF improve the QEPAS sensitivity, which is also immune to environmental acoustic noise. QEPAS has been widely used by research groups that are engaged in trace gas detection in medicine and numerous other applications [14,15,16].

A distinct advantage of the QEPAS technique is its excitation-wavelength independence [17]. This benefit allows the same QEPAS-based trace gas sensor to be used with any type of laser (e.g., the distributed feedback (DFB) diode laser [18], the quantum cascade laser (QCL) laser [19], and the light emitting diode (LED) [20] and any wavelength (e.g., visible [20], near-infrared (NIR) [21], mid-infrared (MIR) [22], and THz spectral region [23]). A gallium antimonide (GaSb)-based interband cascade laser (ICL) became commercially available in 2010 [24]. An ICL is compact and can provide CW radiation, typically between 3.0 μm and 6.0 μm at room temperature operation [25]. Furthermore the ICL size matches the QEPAS-based acoustic detection module (ADM). This wavelength range corresponds to the strongest fundamental vibration band of carbohydrates, which is most suited for optimum detection sensitivity. In this work, we developed a compact QEPAS sensor for C_2_H_6_ detection based on a CW, DFB thermoelectrically cooled (TEC) ICL operating at a wavelength of ~3.3 μm in which an optimum C_2_H_6_ absorption line can be detected.

## 2. Experimental Setup

### 2.1. Absorption Line Selection

**Figure 1:**
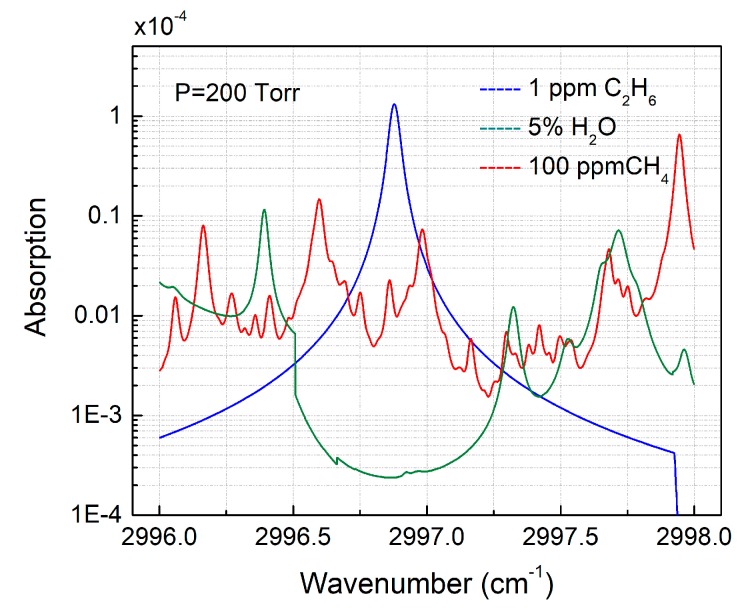
HITRAN based absorption spectra of C_2_H_6_, CH_4_, and H_2_O in a narrow spectral range from 2996 cm^−1^ to 2998 cm^−1^ for specific concentrations of C_2_H_6_, CH_4_, and H_2_O and a 3 cm path length at a pressure of 200 Torr. C_2_H_6_, CH_4_, and H_2_O lines are shown in blue, red, and green, respectively.

C_2_H_6_ has its strong fundamental absorption lines in the mid-infrared spectral range (near 3.3 μm), which permit sensitive and selective detection of atmospheric gases in this spectral range. Within this wavelength region, the potential spectral interference originates mainly from water (H_2_O) and methane (CH_4_). The concentration levels of H_2_O and CH_4_ in air are typically <5% and ~1.8 ppmv, respectively. Therefore, HITRAN absorption spectra of 1 ppmv ethane, 100 ppmv methane, and 50,000 ppmv water at 200 Torr gas pressure and a 3 cm effective optical path length are depicted in Figure 1. The selection of the low gas pressure avoids spectral overlap. A strong C_2_H_6_ absorption line at 3336.8 nm (2996.88 cm^−1^), which is free from spectral interference of other atmospheric gases (such as CH_4_ and H_2_O), was selected as the optimum target absorption line. 

### 2.2. ICL Characteristics

**Figure 2:**
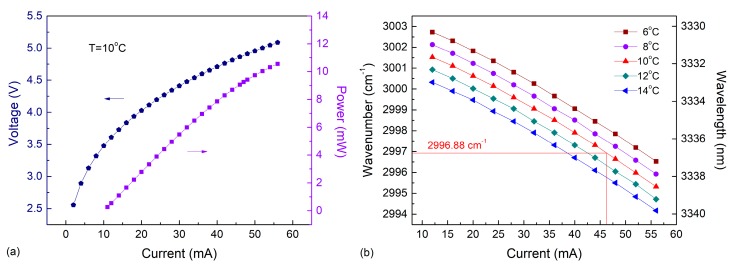
Measured laser characteristics for the 3.3 μm CW TEC ICL at different operating temperatures and injection currents. (**a**) ICL output power and voltage as function of current at 10 °C; (**b**) ICL wavelength (in cm^−1^) as function of ICL current at five temperatures.

A CW TEC interband cascade laser with a wavelength of ~3.3 μm from Nanoplus, GmbH was employed as an excitation source to target the C_2_H_6_ absorption line near 2996.88 cm^−1^. The TO66 mounted ICL was enclosed in a 5 × 5 × 5 cm^3^ cubic heat sink with a TEC. This ICL can be operated at temperatures between 5–15 °C without air or water cooling. The optical power emitted by this laser operated at 10 °C versus five operating temperatures is shown in Figure 2a. With an injected current of 55 mA, the laser power can be as high as ~10.5 mW, which is necessary for a signal-to-noise ratio (SNR) enhancement, since the amplitude of the QEPAS signal is proportional to the laser excitation power. 

At different temperatures of 6–14 °C spaced by 2 °C, the laser wavenumbers versus driving current are shown in Figure 2b. Current and temperature-controlled wavelength tuning coefficients for this ICL were experimentally determined to be −0.141528 cm^−1^/mA and −0.30138 cm^−1^/°C, respectively, larger than those for QCLs. For the available temperature (6–14 °C) and current ranges (10–55 mA), the single frequency spectral tuning range of the laser wavenumber was determined to be 2994.2 cm^−1^–3002.7 cm^−1^. An ICL injection current of 47 mA, combined with a 10 °C operation temperature, was selected for the reported C_2_H_6_ concentration measurements.

### 2.3. Sensor Architecture

**Figure 3:**
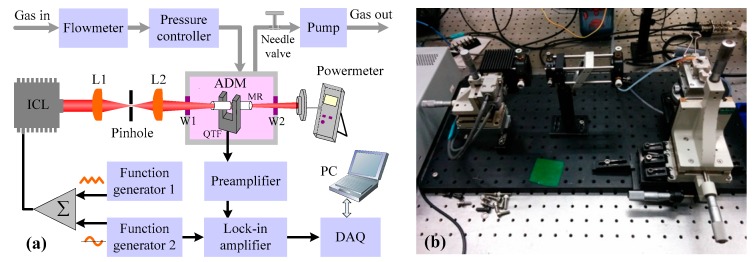
(**a**) Schematic configuration of a 3.3 μm CW, DFB, TEC ICL-based QEPAS system for C_2_H_6_ detection. (**b**) Photo of the optical part of the C_2_H_6_ sensor system.

A schematic of the QEPAS based C_2_H_6_ sensor is shown in Figure 3a. The ICL is equipped with a collimation lens and emits single-mode radiation at a center wavelength of 3337 nm. The collimated beam that exits the TO66 header then passes through a pinhole to reduce the beam diameter due to the fact that the diameter of the original collimated beam is ~6 mm, which is wider than the 300 μm gap between the two prongs of the QTF. The spatial filter consists of two plano-convex CaF_2_ lenses (L1 and L2) with the focal lengths of 5 cm and 4 cm, respectively, and a pinhole with a diameter of 300 μm positioned at the focus position of the two lenses, L1 and L2. The output beam from the spatial filter is directed to an ADM. The ADM includes a standard QTF and two thin metallic tubes with 4 mm length and 0.8 mm internal diameter, which act as acoustic micro-Resonators (AmR) [26]. The ICL beam must be focused through the tubes and the gap between the prongs of the QTF in order to avoid photo-thermal effects and minimize background noise sources [19,27]. A power meter (Ophir, model 3A) is used to monitor the power of the beam after the ADM, verifying that the ICL beam completely passed through the ADM. The ADM is placed in a gas enclosure with a gas inlet and outlet. A pressure controller (MKS Instruments, Inc., USA, model 649) and a vacuum pump are employed to control and maintain the pressure inside the ADM. A needle valve and flow meter are used to control and monitor the gas flow in the sensor system. A photo of the optical system is shown in Figure 3b. 

A triangular wave was used to tune the laser wavelength to scan the absorption line near 2996.88 cm^−1^. Meanwhile, a modulation signal at half of the QTF resonance frequency was applied to modulate the ICL wavelength. The generated electric signal from the QTF was first processed by a pre-amplifier to enhance the SNR and then sent to a lock-in-amplifier for extraction of the 2f signal, whose amplitude represents the C_2_H_6_ concentration. 

### 2.4. Optimization of Modulation Depth

**Figure 4:**
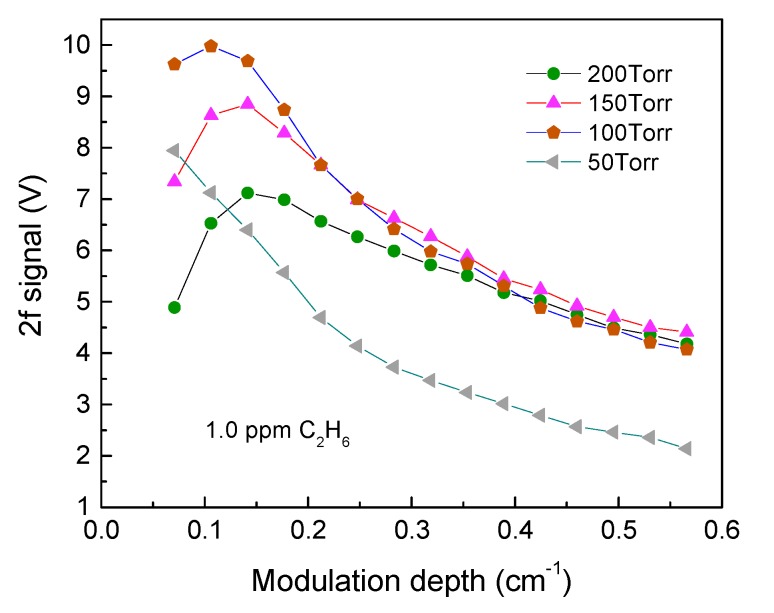
Measured C_2_H_6_ QEPAS signal amplitude as a function of laser modulation depth for a dry 1 ppmv C_2_H_6_:N_2_ mixture at four different pressure values.

In order to obtain the best sensor system detection sensitivity, the gas pressure and modulation depth for wavelength modulation spectroscopy (WMS) should be optimized [28,29]. A certified standard cylinder containing 1 ppmv C_2_H_6_ balanced by UHP N_2_ was employed for the optimization of the ethane sensor system. For each individual pressure ranging from 50 Torr to 200 Torr, the amplitude of the 2f signals was recorded with different modulation depths as depicted in Figure 4. The results demonstrate that the maximum 2f signal of this QEPAS system is observed at 100 Torr and with a modulation depth of 0.106 cm^−1^. For the same modulation depth, the 2f signal amplitude at a pressure of 200 Torr is 34.6% lower than the maximum value. The 2f signal increases with the modulation depth and decreases when the modulation depth is >0.106 cm^−1^.

## 3. Sensor Performance and Discussion

### 3.1. Estimation on SNR

**Figure 5:**
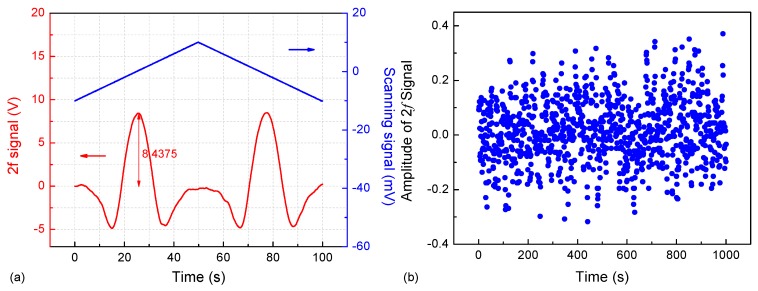
(**a**) Observed 2f signal for a 1 ppmv C_2_H_6_ sample; (**b**) Measured amplitude of the 2f signal by flushing the ADM with pure N_2_ for one hour.

A sinewave signal with a frequency of 16.3 kHz and amplitude of 0.016 V was used to modulate the ICL wavelength, leading to a modulation depth of 0.106 cm^−1^. The driving current and laser temperature were set to 47 mA and 10 °C for the ICL wavelength to be centered at 2996.88 cm^−1^. The pressure in the ADM was set to 100 Torr in order to avoid spectral interference from CH_4_. The signal from the QTF was first sent to the lock-in amplifier and then to a DAQ card. The sampling rate of the DAQ card was set to be 1 kHz. With a 1 s lock-in integration time, the 2f signal was acquired using a triangular wave with a frequency of 0.01 Hz and a peak-to-peak amplitude of 20 mV by scanning the laser wavelength. A spectral scan corresponding to a C_2_H_6_ concentration (1 ppmv) is depicted in Figure 5a. The amplitude of the 2f signal is ~8.44 V. The background noise was measured by flushing the ADM with ultra-high purity (UHP) nitrogen for one hour, as shown in Figure 5b. The noise level (standard deviation for one hour) is ~0.12 V. In this case, the calculated SNR, which was defined as the ratio of the signal amplitude to the 1σ noise level, was ~70.3. A minimum ethane detection sensitivity of 1 ppmv/70.3 ≈ 14 ppbv can thus be estimated. 

### 3.2. Experiment and Results

The sensor linearity was investigated. By diluting a calibration mixture of 1 ppmv C_2_H_6_ with UHP nitrogen, different C_2_H_6_ samples with concentrations ranging from 0 to 1000 ppbv were prepared in order to study the sensor performance. For different C_2_H_6_ samples, the amplitude of the 2f signal (max(2f)) was recorded by implementing line-locking functionality by means of an additional reference channel. The data acquisition time for these measurements was set to 1 s, and the intervals between each C_2_H_6_ concentration value applied to the sensor were set to ~10 min in order to reach a stable level of the measured QEPAS signal.

**Figure 6:**
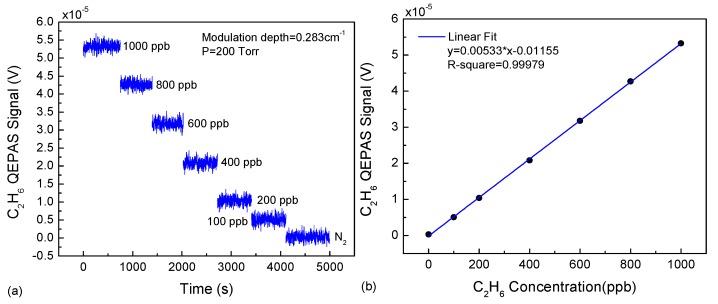
(**a**) QEPAS-based C_2_H_6_ signal at seven C_2_H_6_ concentration levels, ranging from 0 ppbv to 1000 ppbv; (**b**) Linearity of the QEPAS based sensor.

The results of max(2f) for different diluted C_2_H_6_ concentrations are depicted in Figure 6a. Then, the recorded max(2f) and the linear dependence of the averaged 2f signal amplitude as a function of C_2_H_6_ concentration were observed and are plotted in Figure 6b. The calculated R-square value is equal to 0.99979 after a linear fitting procedure, which implies that this QEPAS sensor exhibits excellent linearity for monitoring C_2_H_6_ concentrations. The relationship between the 2f amplitude and the concentration can be expressed as

max(2f) = 0.00533 × C − 0.01155 (V)(1)

in which C is in ppbv. Based on Equation (1), the C_2_H_6_ concentration can be determined using the amplitude of 2f signal. 

**Figure 7:**
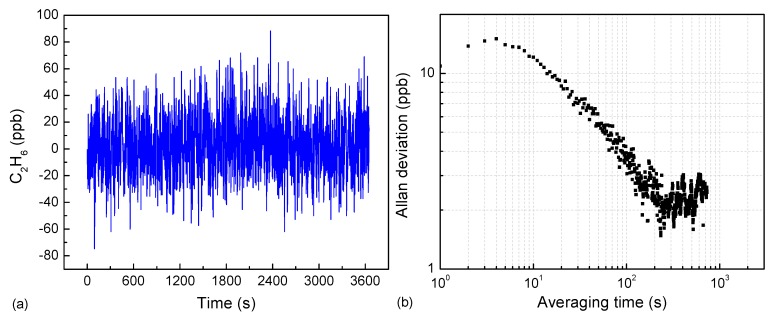
(**a**) Measured C_2_H_6_ concentration by injecting pure N_2_ into ADM; (**b**) Allan-Werle deviation plot for the data shown in Figure 7a.

Allan-Werle deviation measurements were performed to investigate the time stability of the C_2_H_6_ sensor. The C_2_H_6_ sensor operated with pure N_2_ for a period of ~1 h was carried out, and the output results that corresponded to the fluctuation of the sensor output in the absence of the C_2_H_6_ were recorded. An Allan-Werle variance was utilized to analyze the time stability and minimum detection limit (MDL) for this technique. Figure 7a,b exhibits the measured concentration and the Allan-Werle deviation as a function of the integration time t. The plot indicates that the MDL is ~11 ppbv for a 1 s measurement time, as well as ~1.5 ppbv for an optimum integration time of 230 s. The MDL obtained from Allan–Werle plot (11 ppbv) is consistent with the estimation value of 14 ppbv based on SNR and standard deviation measured at 1 ppmv. 

## 4. Conclusions

In this work, we reported the design and results of an innovative, sensitive C_2_H_6_ sensor based on QEPAS. In order to attain ppbv level C_2_H_6_ concentration measurements, a CW, DFB, TEC, ICL with a wavelength of ~3.3 μm combined with a 2f wavelength modulation technique was applied to an interference-free absorption line located at 2996.88 cm^−1^. After appropriate system optimization, the performance of the QEPAS sensor was evaluated using seven C_2_H_6_ samples. An MDL of 11 parts per billion in volume (ppbv) was obtained with a 1s integration time based on the Allan-Werle variance; the MDL could be further improved to ~1.5 ppbv by increasing the integration time up to 230 s. In a future version of this reported C_2_H_6_ sensor, the sensitivity could be further improved by replacing the present ICL with a mid-infrared semiconductor source with higher output power.
